# The Impact of Vitamin D Levels on Clinical Manifestations of Multisystem Inflammatory Syndrome in Children: A Cross-Sectional Study

**DOI:** 10.3390/life13030674

**Published:** 2023-03-01

**Authors:** Davor Petrovic, Benjamin Benzon, Sasa Srsen, Branka Polic, Antonija Vukovic Novogradec, Petra Milic, Josko Markic

**Affiliations:** 1 Department of Pediatrics, University Hospital of Split, Spinciceva 1, 21000 Split, Croatia; 2 School of Medicine, University of Split, Soltanska 2, 21000 Split, Croatia

**Keywords:** vitamin D, hypovitaminosis D, MIS-C, COVID-19

## Abstract

Background: Hyperinflammatory response that resembles Kawasaki disease may develop in children after COVID-19 disease, and it is called multisystem inflammatory syndrome in children. The cause of MIS-C is dysregulated innate immune response and a subsequent cytokine storm that results in endothelial damage. It has been determined that low levels of serum 25(OH)D increase the risk of developing immune-related diseases and disorders. Methods: To determine the incidence of hypovitaminosis D, and a possible correlation between 25(OH)D levels and the clinical severity of MIS-C, 21 patients hospitalized in the University Hospital of Split due to MIS-C were evaluated. Results: Hypovitaminosis D was detected in 95% of MIS-C patients. We found a significant relationship between the severity of MIS-C and 25(OH)D levels, as patients with more severe MIS-C had lower 25(OH)D. MIS-C patients with lower vitamin D levels had worse systolic and diastolic function of the left ventricle according to echocardiograms. There was no relationship between 25(OH)D levels and the tested laboratory inflammatory and cardiac markers. Conclusion: Hypovitaminosis D is very common in children with MIS-C and influences the severity of the disease. VD could be a new potential biomarker in MIS-C, and VD replacement therapy should be considered early on in the treatment of MIS-C.

## 1. Introduction

A new coronavirus was identified in December 2019 in Wuhan, and it reached worldwide pandemic proportions rapidly. The World Health Organization has designated the disease COVID-19 (coronavirus disease 2019) [1]. Children usually develop only mild symptoms; however, in rare cases, a hyperinflammatory response that resembles Kawasaki disease (KD) may develop [2]. That disorder is called multisystem inflammatory syndrome in children (MIS-C) [3], but other acronyms can also be found in the literature [4]. 

The incidence of MIS-C is not yet known, but it appears to be around 2 per 100,000 persons <21 years old [5]. While clinically similar to KD, its epidemiology differs and it mostly affects children older than 5 years of age [4,6]. The usual lag of 2–6 weeks between the peak of COVID-19 and MIS-C cases within communities suggests that MIS-C can be a post-infectious complication rather than acute infection [4]. 

The pathophysiology of MIS-C is not well understood so far [4,7]. Around 35% of affected children have positive SARS-CoV-2 reverse transcription polymerase chain reaction (RT-PCR) results, while positive serology for SARS-CoV-2 is present in 95% of MIS-C patients. Myocardial systolic dysfunction has been described in a large proportion of MIS-C patients. Possible causes of myocardial injury might be systemic inflammation, acute viral myocarditis, hypoxia, stress cardiomyopathy, and, rarely, ischemia caused by coronary artery involvement [8,9]. Around 60% of patients have hemodynamic shock or hypotension from either acute myocardial dysfunction or systemic hyperinflammation and vasodilation [6,9]. Authors suggest that post-viral immunological reaction and systemic hyperinflammation may cause myocardial inflammation and dysfunction in affected children [4,9].

The most common symptoms of MIS-C include fever (99%, with duration of at least 5 days), gastrointestinal symptoms (85%), cardiovascular manifestations (79%), rashes (45–76%), conjunctivitis (30–81%), mucous membrane involvement (27–76%), neurocognitive symptoms (29–58%), and respiratory symptoms (50%) [4,6]. As cardiac involvement is common in MIS-C and a very important prognostic factor, echocardiography is essential in the workup of these patients [4,9,10,11]. Severe disease requiring intensive care was seen in approximately 50% of patients with MIS-C [12]. 

The majority of children with MIS-C are initially treated with both intravenous immune globulin (IVIG) and glucocorticoids. Other interventions depend on the clinical manifestation and the severity of the disease [6,7,9,11,13]. 

In recent years, the impact of vitamin D (VD) on immune and inflammatory response has been intensively researched. Immune cells have vitamin D receptors (VDRs) and the ability to bind VD, and through that mechanism VD influences immune response and the proliferation and differentiation of immune cells [14,15,16,17,18]. It has been determined that low levels of serum 25-hydroxyvitamin D (25(OH)D) increase the risk of developing immune-related diseases and disorders [19]. 

The aim of this study was to determine the incidence of hypovitaminosis D in patients with diagnosed MIS-C. Additionally, a possible correlation between 25(OH)D levels and the clinical severity of MIS-C estimated by clinical presentation, laboratory markers, and echocardiograms was evaluated.

## 2. Materials and Methods

### 2.1. Study Population

Participants were patients hospitalized in the Department of Pediatrics at the University Hospital of Split and discharged with a diagnosis of MIS-C in the period from 1 June 2020 until 31 May 2022. Eligible participants were included consecutively. MIS-C diagnosis was confirmed by clinical examination, laboratory results, and echocardiograms according to the guidelines [20,21,22]. 

This study was approved by the Ethics Committee of the University Hospital of Split (approval code: 500-03/21-01/32; approval date: 26 February 2021). Written informed consent was provided by the participants’ parents. Patients whose parents rejected the offer to participate in the study and patients with missing data were excluded.

For the creation of this paper, STROBE guidelines (STrengthening the Reporting of OBservational studies in Epidemiology) were used [23].

### 2.2. Variables 

The following parameters were evaluated: 25(OH)D level, procalcitonin (PCT), C-reactive protein (CRP), erythrocyte sedimentation rate (ESR), fibrinogen, D-dimer, ferritin, absolute neutrophile count (ANC), absolute leucocyte count (ALC), thrombocyte and erythrocyte count, interleukin 6 (IL-6), troponin T, N-terminal proBrain Natriuremic Peptide (NT-pro-BNP), albumins, total proteins, liver enzymes, lactate dehydrogenase (LDH), and electrolytes (sodium, calcium, potassium, chloride, phosphate, and magnesium). We also collected data on gender and patients’ age at admission. Medical records were used to collect data. Admission into the PICU was based on clinical and hemodynamic status at presentation of MIS-C according to the PICU admission criteria: shock, hemodynamic instability, arrhythmia, ill-appearing, significant respiratory compromise, evidence of organ dysfunction/injury, requiring respiratory or cardiovascular support [13].

### 2.3. Methods

Peripheral venous blood was sampled in BD Vacutainer^®^ SSTII Advance (BD, Plymouth, UK) tubes and sent for analysis to the Department of Medical Laboratory Diagnostics at the University Hospital of Split. Serum concentrations of 25(OH)D are considered the best measure of vitamin D status [24]. The 25(OH)D level in the serum was analyzed by using an Elecsys Vitamin D total assay with a Cobase601 analyzer (Roche Diagnostics International Ltd., Rotkreuz, Switzerland). As sufficient 25(OH)D levels were considered to be concentrations >75 nmol/L, VD insufficiency was diagnosed when 25(OH)D levels were between 50 and 75 nmol/L, while deficiency was diagnosed with 25(OH)D levels <50 nmol/L [25,26].

Patients with MIS-C underwent full cardiac evaluation, including an ECG and transthoracic echocardiogram (TTE). According to the recommendations for cardiovascular imaging of patients during the COVID-19 pandemic, standard two-dimensional echocardiography was performed by experienced cardiac sonographers with a GE Vivid S70N Ultrasound System (GE Healthcare, Chicago, IL, USA) [27,28,29]. 

Echocardiogram findings included cardiac function, coronary artery abnormalities, cardiac valve regurgitation, and pericardial effusion. The standard echocardiographic parameters included were the left ventricle ejection fraction (LVEF) assessed with two methods (the Simpson’s biplane and M-mod methods); spectral Doppler mitral inflow peak velocities; early diastolic septal and lateral mitral annular peak velocities assessed by TDI; tricuspid annular plane systolic excursion (TAPSE); and lateral tricuspid annular peak velocity assessed by TDI (TAPSV). Coronary arteries were assessed according to the AHA Guidelines for Kawasaki disease [30], and the abnormalities were classified using the Boston z-score system. Analysis of left ventricle longitudinal strain (LS) was not performed due to technical difficulties.

According to the severity of the disease, MIS-C patients were assigned into three groups: mild, moderate, and severe MIS-C [4,31]. Patients with initial borderline clinical and laboratory criteria for the diagnosis of MIS-C, with normal vital signs apart from fever, with only mild symptoms suggestive of MIS-C, with mild dehydration, who were well-appearing, and who had no signs of myocarditis or heart failure in echocardiography results were included in the mild MIS-C group. Later on, during their hospitalization, they met definite criteria for the diagnosis of MIS-C. The moderate MIS-C group included patients that immediately met the MIS-C case definition but did not present symptoms of shock or other PICU admission criteria. The severe MIS-C group included patients that met the case definition and the PICU criteria. 

To better differentiate Kawasaki disease and MIS-C, as well as to avoid false positive MIS-C patients, special attention was paid to the distinctions between MIS-C and Kawasaki disease according to the literature: history of documented or suspected COVID-19 prior to the MIS-C symptoms, age at presentation of the disease, results of testing for SARS-CoV-2 (PCR and serology), the existence of gastrointestinal symptoms, myocardial dysfunction, shock, coronary aneurysms, and levels of inflammatory markers (especially CRP, ferritin, D-dimer, absolute lymphocyte, and platelet counts) [4].

Data presented in this section were obtained before starting the treatment of MIS-C and hypovitaminosis D. 

### 2.4. Statistics

Descriptive data were shown as a median value with a minimum and maximum. The relationship between VD levels and other variables was modeled by linear or nonlinear regression, or in some cases *t*-tests. The size of an effect is presented as a point estimate and 95% confidence interval of model coefficient or difference. As statistical measures of evidence, in addition to a 95% CI, R^2^ and p values were also used. P values were interpreted in accordance with the American Statistical Association (ASA) Statement on Statistical Significance and P-Values. Statistical analysis was performed using GraphPad software (La Jolla, CA, USA). 

## 3. Results

During the study period, a total of 25 patients diagnosed with MIS-C were eligible for inclusion in the study. Of these, four of them (16%) were excluded due to missing data (lack of a 25(OH)D level value). In total, 21 participants remained in the study for further statistical analysis. 

Two thirds of the participants were males (14; 67%), and one third were females (7; 33%). COVID-19 rapid antigen tests were negative in all of our patients. Medical history of documented or suspected COVID-19 indicated previous infection in 76% of our patients, with an average duration of 41 days between SARS-CoV-2 infection and the onset of MIS-C. Positive serology for SARS-CoV-2 was found in 18 patients (86%). According to clinical presentations, there were five mild (24%), eight moderate (38%), and eight (38%) severe MIS-C patients. Systolic dysfunction and symptomatic myocarditis were diagnosed in 33% of our patients. Most of the severe MIS-C patients (6; 75%) had cardiac involvement. We had no patients with coronary artery dilatation or aneurysm. VD insufficiency was diagnosed in 5 patients (24%), and VD deficiency in 15 patients (71%). Only one patient (5%) had a sufficient level of 25(OH)D.

Detailed data about MIS-C patients laboratory parameters, LVEF, and 25(OH)D levels can be found in Table 1.

**Figure 1:**
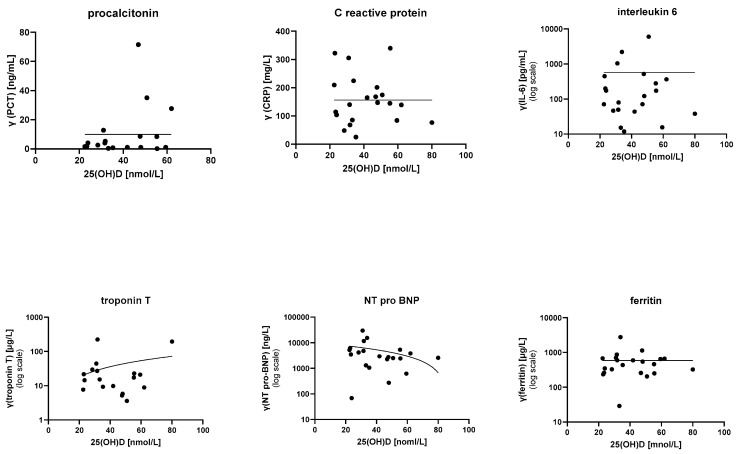
Relationships between 25(OH)D levels and various inflammatory and cardiac markers. Statistical modeling did not show any relationships. PCR—procalcitonin; CRP—C-reactive protein; IL-6—interleukin 6.

The data did not show any conclusive relationship between 25(OH)D levels and tested laboratory parameters (PCT, CRP, ESR, leucocyte count, lymphocyte count, platelet count, erythrocyte count, fibrinogen, D-dimer, IL-6, ferritin, albumin, total proteins, liver enzymes, electrolytes, troponin T, and NT-pro-BNP). Inflammatory and cardiac markers are depicted in Figure 1.

**Figure 2:**
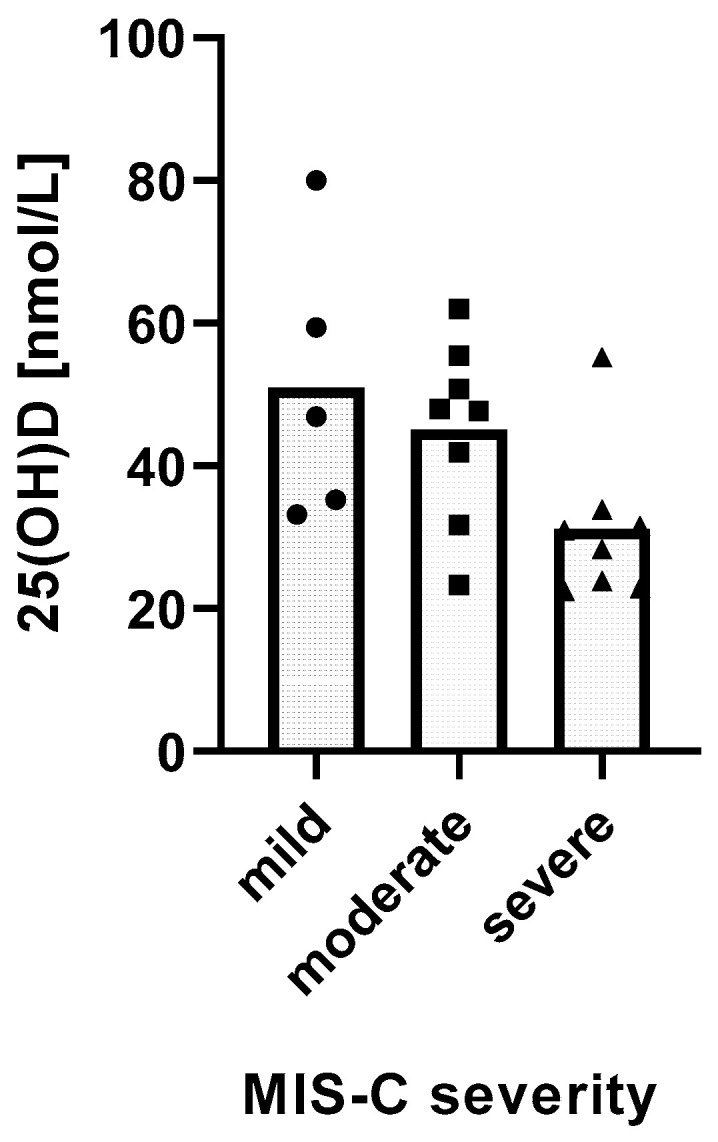
Relationship between 25(OH)D levels and the severity of MIS-C. Distribution of mean 25(OH)D levels in groups of MIS-C patients with different disease severity. Test for linear trends: β = −10.26 nM/severity category, 95% CI −18.38 to −2.13, *p* = 0.0162, R^2^ = 27.62%.

We found a significant relationship between the severity of MIS-C and 25(OH)D levels. MIS-C severity was inversely and linearly related with 25(OH)D levels (*p* = 0.0162) (Figure 2). 

**Figure 3:**
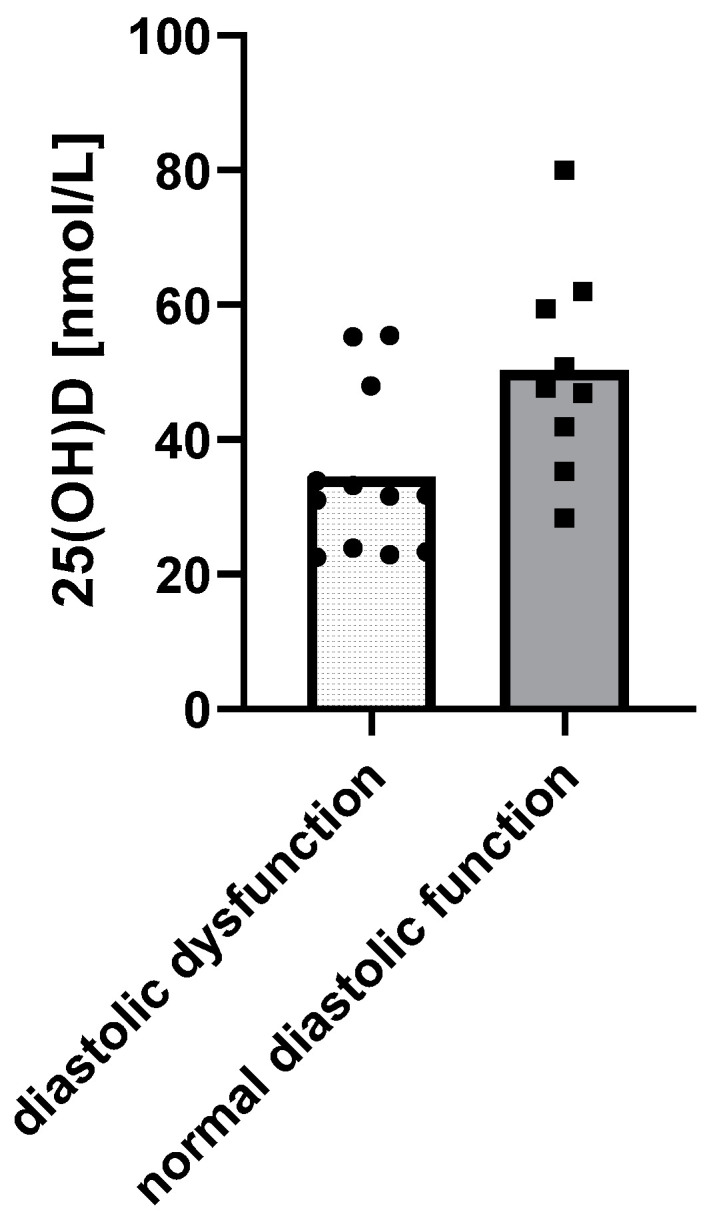
Relationship between 25(OH)D levels and the diastolic function of the left ventricle using a *t*-test: Δ = 15.85 nM, 95% CI 3.3 to 28.37, *p* = 0.0158, R^2^ = 26.99%.

When it comes to cardiac function, children with diastolic dysfunction of the left ventricle in echocardiograms had lower 25(OH)D levels (*p* = 0.0158) (Figure 3). 

**Figure 4:**
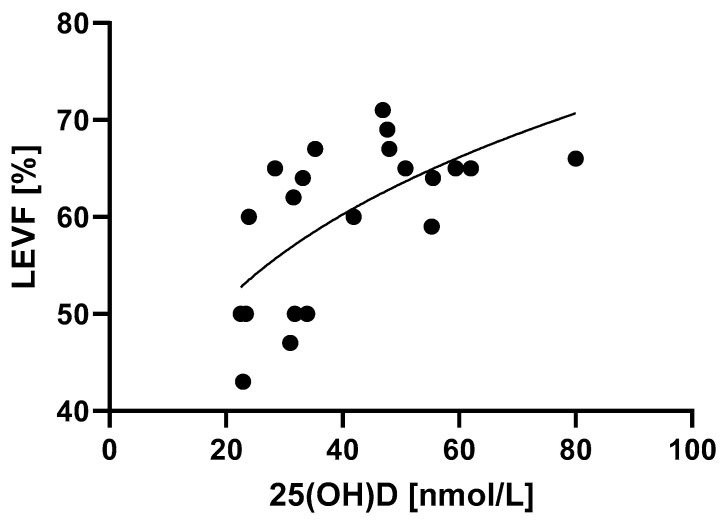
Relationship between 25(OH)D levels and the systolic function of the left ventricle determined by LVEF. *p* = 0.0018, R^2^ = 40.9%.

Left ventricle ejection fraction was associated with 25(OH)D levels in a manner that is best described by power law (Figure 4, Table 2) (*p* = 0.0018). MIS-C patients with higher 25(OH)D levels had better systolic function of the left ventricle, which was determined by higher LVEF values. 

## 4. Discussion

The patient demographic data analyzed in this study were comparable with other studies regarding MIS-C. Similar median age (8 years) and male predominance was found in large systematic reviews [4,6].

Common laboratory abnormalities noted in patients with MIS-C, such as lymphocytopenia, neutrophilia, anemia, thrombocytopenia, elevated CRP, ESR, D-dimer, fibrinogen, ferritin, PCT, IL-6, troponin T, NT-pro-BNP, lactate dehydrogenase, liver enzymes, hypoalbuminemia, and hypertriglyceridemia [3,4,6,11], were also noted in our patients.

In this study, 33% of patients had significant cardiac involvement during MIS-C. Other authors also described left ventricular systolic dysfunction in 35–100% of patients and symptomatic myocarditis in 40–80% of patients diagnosed with MIS-C [3,9]. Coronary artery dilation or aneurysms were described in 6–24% of patients, and arrhythmias were observed in 12% of MIS-C patients [6,9,10]. However, those complications were not described in our patients.

Intensive care admission of MIS-C patients is common (60–80%), with a median duration of 4 days and a median hospitalization time of 8 days. Deaths are rare and occur in 1.7–1.9% of patients, but overall prognosis of MIS-C looks positive as most children have a full clinical recovery [6,13]. More than a third of our patients (38%) had severe MIS-C and were admitted and treated in the PICU, which is a lower level of incidence than in other studies. Additionally, all of our patients survived.

We found hypovitaminosis D in almost all of our MIS-C patients (95%), and the majority of patients (71%) had VD deficiency. Median 25(OH)D level in our patients was 35.3 nmol/L. That was a higher level of incidence for hypovitaminosis D than in a similar study by Hadžić-Kečalović et al., where the authors also found a high incidence of hypovitaminosis D in MIS-C patients (78% of the patients) [32]. In Turkey, a land with a similar Mediterranean geographical origin, Ekemen Keles et al. reported an almost identical median 25(OH)D level (36.4 nmol/L) to the one in our study. Additionally, they found VD insufficiency in 74% of their MIS-C patients, though they used a lower cutoff point for the diagnosis of VD insufficiency (50 nmol/L) than in our research. They also reported that MIS-C patients had significantly lower VD levels than participants in the control group [33]. A similar high incidence of hypovitaminosis D in pediatric MIS-C patients with a predominance of VD deficiency was found in other studies of different geographical origin [34,35].

The pathophysiology of MIS-C is not well understood, but there is evidence of a dysregulated innate immune response and a subsequent cytokine storm that results in endothelial damage. Some authors suggest that pathophysiological mechanisms in MIS-C include persistent immunoglobin G (IgG) antibodies, T cell-mediated cell damage, or the activation of inflammation. Antibodies and T cells attack cells expressing viral antigens or attack host antigens which cross-react or mimic viral antigens [4,7].

Recently, there has been a lot of emphasis on VD’s influence over immune and inflammatory response. VD influences the immune system in a variety of ways: directly with immune cells through VDR [14], as a selective regulator of lymphocyte activation and proliferation, through monocyte differentiation in promyelocytes, and as a modifier of cytokine synthesis in immune cells [15,16,17,18]. VDR was found on antigen-presenting immune cells, such as T lymphocytes, dendritic cells, and macrophages [36,37,38,39,40,41]. VD even modulates humoral immune response by influencing the proliferation and production of immunoglobins in B lymphocytes, as well as the differentiation of B lymphocyte precursors into plasma cells [42]. It has been demonstrated that supplementation with VD is beneficial for Th1-mediated autoimmune response [43], and that VD insufficiency turns immune response towards loss of tolerance [38,44].

We have found a significant relationship between the severity of MIS-C and 25(OH)D levels. Patients with severe MIS-C had lower 25(OH)D levels, with very low median 25(OH)D levels (29.7 nmol/L). Darren et al. also found that patients with severe MIS-C treated in the PICU had lower mean 25(OH)D levels compared to the non-PICU group; however, the relationship was not statistically significant (19.5 v. 31.9 nmol/L; *p* = 0.11). In their study, a significant association between 25(OH)D levels and MIS-C severity in children was not found, though they concluded that their study was not adequately powered to evaluate that relationship [34]. Rivera et al. found that 90% of patients with severe VD deficiency had severe MIS-C, and they point out a potential association between 25(OH)D levels and MIS-C severity [35]. Other authors also reported that 25(OH)D levels correlate with MIS-C severity. They observed that the number of ≥4 affected organ systems was significantly higher in the group with inadequate 25(OH)D levels. Additionally, they pointed out that patients treated in the PICU had significantly lower 25(OH)D levels than patients in the non-PICU group, and that all of the patients with hypotension and signs of shock had inadequate 25(OH)D levels [33]. There is a connection between Kawasaki disease, a disease with very similar pathophysiology to MIS-C, and VD mentioned in the literature. Meyer et al. found that VD supplementation lowered the occurrence of Kawasaki disease in a German population [45]. Korean authors stated that patients with VD deficiency showed statistically significant resistance to intravenous immunoglobulin [46].

There are no studies that have evaluated the pathophysiological mechanism through which VD influences the development of MIS-C. There are, however, studies that confirm a correlation between VD status and COVID-19 severity and mortality in adults. Many reviews suggest that the state of out-of-control release of a variety of inflammatory cytokines (“cytokine storm”) is one of the main hallmarks of COVID-19 severity. Observational studies showed elevated levels of different cytokines in COVID-19 patients, such as TNFα, IL-1β, IL-6, IL-8, IL-10, and IL-17 [47,48]. Experts state that VD promotes the production of antimicrobial and antiviral proteins such as β-defensin 2 and cathelicidin, and that it also suppresses cytokine storms and inflammatory processes in COVID-19 [49]. As VD has been shown to have immunomodulatory activity on IL-6, and anti-IL-6 agents have already demonstrated a role in the course of COVID-19, it has been speculated that administration of VD supplementation could be beneficial in terms of COVID-19 progression [47,50]. In the case of MIS-C, some authors conclude that VD probably plays an immunomodulatory role by influencing immunocomplex-mediated immune response and by changing the levels of specific cytokines during development of the disease [51,52]. VD affects Th cell polarization and consequently produces specific anti-inflammatory cytokines [53], a mechanism which could limit the production and damaging effect of pro-inflammatory cytokines during MIS-C [51].

We did not find any correlation between 25(OH)D levels and individual inflammatory markers. Similar results were found in other studies as well [32,35]. We can assume that inflammatory markers cannot be used individually as a marker for MIS-C severity; however, they are useful for the evaluation and stratification of the disease when observed together with other clinical parameters.

We found a significant relationship between 25(OH)D levels and cardiac presentations of MIS-C. Our patients with lower 25(OH)D levels had more frequent LV diastolic dysfunction along with lower LVEF values. Rivera et al. also found that MIS-C patients with severe vitamin D deficiency had an increased risk of cardiac involvement. They concluded that severe vitamin D deficiency predisposes patients to cardiovascular involvement [35]. Cardiac involvement has been recognized as an important factor in the clinical presentation and progression of MIS-C. Cardiac dysfunction may present with hemodynamic compromise and can develop arrhythmias during their course of illness, which can seriously change the progression of the disease. In the literature, early cardiac evaluation is recommended in suspected MIS-C cases [9,13,29]. The role of VD in cardiovascular pathophysiology has been studied extensively, and hypovitaminosis D is a common finding in patients with cardiovascular disease. It has been postulated that VD plays a big role in endothelial function. VDRs are expressed in many cells and tissues of the cardiovascular system, including vascular smooth muscles, endothelia, and myocardia [54,55]. Margossian et al. found that vitamin D deficiency is associated with lower LV mass z-scores [56]. McNally et al. demonstrated that VD deficiency was associated with prolonged mechanical ventilation and ICU stays after pediatric cardiac surgery [54]. The authors surmise that patients with VD deficiency have non-ideal baseline endothelial function, oxidative stress, membrane transport, and cell matrix homeostasis, which puts them at higher risk of significant cardiovascular involvement and predisposes them to myocardial malfunction, endothelial dysfunction, peripheral edema, shock, pulmonary edema, and hypotension, all of which are clinical manifestations of severe cardiac involvement and illness due to MIS-C [35].

There are some authors that suspect hypovitaminosis D to be a consequence of MIS-C [57]. They suggest that VD could be one of the biomarkers of MIS-C. Greater inflammatory processes need more of the active form of VD for the appropriate anti-inflammatory role, resulting in reduced overall VD levels. With this in mind, low levels of VD in severe inflammatory diseases could be the result of the severity of the disease and not a predisposing factor. Further, low VD levels are reported in a wide range of disorders because the inflammatory reactions involved in the process of the diseases can reduce 25(OH)D [51,58]. Nevertheless, the authors conclude that hypovitaminosis D should be treated in order to promptly raise 25(OH)D levels, which can potentially contribute to reducing the severity of MIS-C in certain circumstances [51]. Estrada-Luna et al. conclude that, in addition to the main pharmacological treatment for MIS-C (IVIG and steroids), complementary therapy based on natural compounds such as VD has shown promising results [59]. Our patients with newly diagnosed hypovitaminosis D were treated with peroral VD according to Global Consensus Statement guidelines [60].

Limitations of the study: This was a single-center study where we recruited patients from the coastal part of Croatia; thus, there is a chance of selection bias based on the geographical origin of the patients. Additionally, we had a relatively small sample size, which needs to be increased in future studies.

## 5. Conclusions

Hypovitaminosis D is very common in children with MIS-C and influences the severity of the disease. There is a correlation between 25(OH)D levels and the intensity of the cardiac presentations of MIS-C. VD plays an important immunomodulatory role in the development of MIS-C, as in other autoimmune diseases. VD could be a new potential biomarker in MIS-C, and VD replacement therapy should be considered during the early stages of the treatment of MIS-C. To further investigate the connection between VD and MIS-C, new studies with larger sample populations are needed, especially randomized controlled studies.

## Figures and Tables

**Table 1:** Distribution of age, 25(OH)D, laboratory parameters, and LVEF at the presentation of MIS-C.

	All Patients (N = 21)			Mild MIS-C (N = 5)			Moderate MIS-C (N = 8)			Severe MIS-C (N = 8)		
	Median	Min.	Max.	Median	Min.	Max.	Median	Min.	Max.	Median	Min.	Max.
Age, years	8.08	1.25	16.58	6.25	3.58	14.08	8.33	1.25	16.58	9.92	3.25	14.58
25(OH)D, nmol/L	35.3	22.5	80	46.9	33.2	80	47.85	23.4	62	29.7	22.5	55.3
PCT ^a^, ng/mL	2.135	0.18	>100	0.905	0.39	71.55	3.485	0.18	35.1	3.285	1.6	>100
CRP ^b^, mg/L	142.6	25	339.7	84.3	25	168.2	147	68	339.7	177.2	48.1	322.5
IL-6 ^c^, pg/mL	134.9	11.93	6026	15.62	11.93	71.55	173.4	43.77	6026	227.2	46.77	2218
Ferritin, ng/mL	449	29	2767	325	29	647	545	205	1139	570.5	235	2767
D-dimers, mg/L	3.465	1.27	22.9	2.42	1.27	3.18	4.3	1.52	16.66	5.315	1.61	22.9
Fibrinogen, g/L	5.1	2.3	>9	5.1	3.3	6.1	5.1	4	>9	5.3	2.3	6.1
Troponin T, ng/L	17.6	<3	2122	15.3	<3	196	9.8	3.6	224	24.45	7.7	2122
NT-pro-BNP, pg/mL	3063	274	29766	1311	626	2583	2967	274	11681	5303	2416	29766
Leucocytes, G/L	6.5	2	14.9	7	6	14.9	4.9	2	16	8.15	3.6	10.7
Platelets, G/L	185	67	793	176	112	793	178	72	278	202.5	67	380
LVEF ^d^, %	61	43	71	66	64	71	60	50	69	54.5	43	65

^a^ procalcitonin; ^b^ C-reactive protein; ^c^ interleukin 6; ^d^ left ventricle ejection fraction on echocardiogram; Min—minimum; Max—maximum.

**Table 2:** Model describing the relationship between EF and 25(OH)D.

Model Equation	Model Parameter	Point Estimate	95% CI
$\mathrm{LVEF}\left(\%\right)=b\cdot c{\left[25\left(\mathrm{OH}\right)D\right]}^{\frac{1}{a}}$	b	25.73	15.59 to 42.10
	a	4.33	2.74 to 10.23

Legend: LVEF—left ventricle ejection fraction, c [25(OH)D]—molar concentration of 25-hydroxyvitamin D3.

## Data Availability

The data that support the findings of this study are available from the corresponding author upon reasonable request.
